# Innovations in Immunosuppression for Intestinal Transplantation

**DOI:** 10.3389/fnut.2022.869399

**Published:** 2022-06-15

**Authors:** Harween Dogra, Jonathan Hind

**Affiliations:** Pediatric Hepatology, Gastroenterology and Nutrition Center, King's College Hospital, London, United Kingdom

**Keywords:** intestinal failure, bowel transplant, parenteral nutrition, immunosuppression, tissue engineering

## Abstract

It has been 57 years since the first intestinal transplant. An increased incidence of graft rejection has been described compared to other solid organ transplants due to high immunogenicity of the bowel, which in health allows the balance between of dietary antigen with defense against pathogens. Expanding clinical experience, knowledge of gastrointestinal physiology and immunology have progress post-transplant immunosuppressive drug regimens. Current regimes aim to find the window between prevention of rejection and the risk of infection (the leading cause of death) and malignancy. The ultimate aim is to achieve graft tolerance. In this review we discuss advances in mucosal immunology and technologies informing the development of new anti-rejection strategies with the hope of improved survival in the next generation of transplant recipients.

## Introduction

For those patients with irreversible intestinal failure, and significant complications including very poor quality of life, bowel transplant offers the only possibility to achieve enteral autonomy (1).

Bowel transplants have been attempted since the 1960's but success was limited by challenges in balancing prevention of rejection with risks of infection and malignancy. Advances in immunosuppression therapies significantly improved outcomes, the introduction of ciclosporin first described for use after bowel transplant by Grant et al. (2), and then more effective calcineurin inhibitors (3). However, survival rates after intestinal transplant currently lag behind those of isolated liver or renal transplant. Better graft survival after isolated small bowel transplant is associated with fewer, less severe, episodes of rejection (4) and liver-inclusive grafts are shown to have reduced rates of rejection compared to bowel transplant alone (though with higher rates of GvHD) (5, 6).

The Intestinal Transplant Registry and published single center experiences describe patient survival at 55–66% at 5 years and graft survival at 48–62%, with rejection (13%) and sepsis (50%) being the biggest contributors to patient death (7, 8). This is compared to a 5 year survival of 95% reported after liver transplant (9). Registry data reports PTLD (post transplant lymphoproliferative disorder) occurrence as 8% in the current era (10).

Can new scientific advances support improved management strategies to allow intestinal transplant survival to match those for other solid organ transplants, particularly in management of rejection, infection and malignancy?

Studies of mucosal immunology in other bowel disorders, such as inflammatory bowel disease (IBD), have revealed a complex network of interactions between immune and GI tract cell types. Developments in molecular and microscopy techniques allow increasingly detailed descriptions of gut specific immune cell subsets. Enhancing those subsets which limit inflammatory responses is advancing, with engineered monoclonal antibody and small molecule therapies already being used to manage other inflammatory disorders of the gut. Research on the gut microbiome has exploded since the first intestinal transplants, however, it remains to be proven whether the microbiome can be manipulated to alter mucosal immune responses. Developing these approaches may allow us to replicate the physiological balance of immunogenicity and tolerogenicity within the bowel after transplant, enhance immune monitoring and immunosuppression and thus improve transplant outcomes. Tissue engineering is also an expanding field, organoids from autologous or animal derived cells could become part of the future of intestinal transplant, potentially preventing the need for immunosuppression altogether.

## Immune Cell Modulation in Bowel Transplant

Rejection and infection management strategies can be improved with better understanding of the immune interactions we aim to modulate. Relevant lymphocyte sub sets in the gut are the regulatory T cells (T_reg_), resident memory (T_rm_) and effector memory cells (T_em_) which themselves can be derived from CD4 or CD8 progenitors. High concentrations of T_reg_ have been demonstrated in the bowel, as their name suggests, to allow tolerogenicity toward nutrient antigen and the healthy microbiome (11). T_reg_ are characterized by the FOXP3 nuclear transcription factor (IPEX is a human disease where there is a mutation in the *FOXP3* gene, characterized by immune dysregulation, demonstrating the important role of these cells in regulating or immune system). T_reg_ can also be characterized by cell surface markers CD45 and CD25 (a component of the IL-2 receptor, which is targeted by basilixumab). T_reg_ are shown to suppress CD4 and CD8 T cells, possibly also B cells. T_regs_ can also produce anti-inflammatory cytokines TGF-beta and IL-10 (12).

There is thought to be a plasticity in phenotype, with peripheral T_reg_ cells migrating to the intestine and *via* interactions with other immune cell types and antigens being able to shift to express markers more associated with CD4 T_em_ cells (13). Studies in different solid organ transplant models demonstrate a role for donor specific T_reg_ in promoting graft tolerance.

Studies in Pediatric liver transplant recipients have described a role for T_reg_ in episodes of acute rejection (14).

### T Cell Chimerism Role in Graft Rejection

Chimerism after organ transplant refers to the presence of donor/graft and recipient/host immune cells within the organ graft. It is the interaction between the donor and recipient immune systems which modulates between episodes of cellular rejection and tolerance. Populations of distinct tissue resident (rather than circulating) T cells have been demonstrated in the human GI mucosa. The unique environment of bowel transplant, with populations of host and donor cells, has allowed this tissue resident cell type to be directly interrogated. Bartolome-Casado et al. (15) have demonstrated CD8 T_rm_ cells not only persist from the donor in the intestinal mucosa but are also functional. In the parallel paradigm of lung transplant, Snyder et al. (16) have demonstrated that host T cells can migrate into the mucosa to acquire a similar tissue resident phenotype.

Donor chimerism in the intestinal mucosa after bowel transplant has been observed to persist up to 5 years post-transplant (17). Zuber et al. (18) found early T cell mediated rejection after bowel transplant demonstrated a higher proportion of host vs. graft T cell clones, which declined after resolution, compared to little difference in late episodes of rejection. They described persistence of a stable repertoire of donor T cells in non-rejection bowel transplant mucosa. Also, a high turnover of recipient T cells migrating into the graft at early time points which then stabilized and replaced the donor T cells. In a separate study Zuber et al. (19) described macrochimerism of immune cells, i.e., >1%, between donor and recipient may be predictive of inducing tolerance. Their study demonstrated chimerism in peripheral blood of intestinal transplant patients, especially T cell chimerism, was associated with fewer/less severe episodes of rejection (similar findings were demonstrated in the lung transplant paradigm by Bartolome-Casado). Lower severity of rejection was also associated with lower levels of pre-formed donor specific antibodies (DSA). Better levels of chimerism were demonstrated after multivisceral rather than isolated small bowel transplant (possibly related to splenectomy and thus reduced host lymphoid compartment in the former). In the future, routinely measuring chimerism within the intestinal graft may become a model for predicting episodes of rejection and altering immunosuppression to prevent it.

### Immunosuppression Regimens to Prevent Acute Cellular Rejection

Trafficking signals in the mucosal microenvironment attract effector cells of innate immunity such as neutrophils, circulating lymphocytes and macrophages *via* cell surface receptors and adhesion molecules. Cytokine intermediaries, such as IL-2, IL-23, TNF-alpha can be released and promote pro-inflammatory signals between tissue resident lymphocyte sub-sets and their neighbors. These pathways can all be targeted to modulate immunosuppression post-transplant.

Initial use of calcineurin inhibitor cyclosporin in the 80's, was replaced by tacrolimus (FK506) in the 90's as primary maintenance due to better outcomes and improved side effect profile. The most recent annual report from the US Organ procurement and transplant network in 2020 states Tacrolimus remains the choice for maintenance immunosuppression after bowel transplant, in combination with corticosteroids (inhibit T cell proliferation, antibody production and neutrophil activity) and/or mycophenolate mofetil (inhibitor of purine synthesis) (20, 21). Tacrolimus targets the intracellular calcineurin signaling pathway (3) to inhibit T cell proliferation. Sirolimus is also used as an adjunct immunosuppressant, but with the disadvantage of delayed wound healing (22, 23), it inhibits T cell proliferation by blocking the action of the intra-cellular signaling enzyme mTOR (mammalian target of rapamycin).

Induction immunosuppression, to deplete T cells pre/intra-operatively, is widely used, with rabbit ATG (anti-thymoglobulin), alemtuzumab (anti-CD52 antibody targeting lymphocyte cell surface receptor), muromonab CD3 (antibody against T cell surface protein CD3) or daclizumab (antibody against cell surface receptor CD25), in order of most to least used as reported in the US in 2008 (21). Basilixumab (antibody against IL-2) is used as an induction agent in some centers (22, 24).

Recent single center experiences describe analysis of induction regimens with the aim to optimize outcomes. Devine et al. (25) have reported a higher occurrence of post-transplant lymphoproliferative disorder (PTLD) in pediatric intestinal transplants recipients receiving basilixumab (anti-IL-2) and alemtuzumab (anti-CD52) compared to rATG (rabbit anti-thymoglobulin). Vianna et al. (26) have reported alemtuzumab induction alone lowered incidence of GvHD (Graft versus Host Disease), but also that intensive induction with combined rATG and rituximab (anti-CD20) was protective against episodes of acute rejection in the early post-transplant period. These studies demonstrate the balance between graft and host that needs to be achieved for graft survival and reduced risk of malignant host immune proliferation (Table 1).

**Table 1:** Summary of risks and benefits of new induction regimes pre-intestinal transplant.

**Induction regime**	**Mode of action**	**Risk/benefit**	**References**
Basilixumab and Alemtuzumab	Anti IL-2 and anti CD52	Increased risk of PTLD	(25)
Alemtuzumab	Anti CD52	Lower risk of GvHD	(26)
rATG and Rituximab	Anti thymoglobulin and anti CD20	Lower risk of ACR	(26)

Immunosuppression strategies such as that described by Ceulemans et al. (27) use knowledge of immune responses to optimize outcomes. The Leuven protocol, with donor-specific blood transfusion and lower long-term maintenance immunosuppression aimed to induce T_reg_ cells thus promoting graft tolerance, and indeed had good graft and patient outcomes. Of note in this and other series (28) there was strict donor selection and short cold ischemic time in order to reduce the inflammatory stimulation of ischemia reperfusion injury.

## Proposed New Drug Therapies

### Cytokine Directed Therapies

Increased expression of TNF alpha has been described in episodes of small bowel transplant rejection (29). Pech et al. have described use of anti-TNF therapy to overcome episodes of acute rejection in a rat model of bowel transplant (30) and also reduce inflammatory infiltrate and dysmotility in the same rat model (31). Case reports and cohort studies describe beneficial use of anti-TNF to treat patients with acute rejection resistant to conventional therapy, and one report describing use for mucosal inflammation in an intestinal graft (32–35). Kroemer et al. (36) have demonstrated a good response to infliximab therapy in episodes of rejection unresponsive to ATG. When immune cells from the intestinal mucosa of these patients were interrogated, non-responders to ATG were shown to have a higher proportion of IL-17 and TNF-alpha producing CD4 T cells. The authors hypothesize this is why targeted therapy with anti-TNF therapy infliximab showed endoscopic and histological resolution of rejection.

Kodama et al. (37) used a rat model of intestinal transplant to investigate the novel immunosuppressive compound Prenylated Quinolinecarboxylic Acid compound 18 (PQA-18). The compound inhibits p21-activated kinase 2, reducing cytokine production of IL-2, IL-6 and TNF-alpha. The study showed better graft survival associated with use of PQA-18 likely secondary to suppressed T cells and macrophage differentiation.

### Anti-cell Adhesion Molecules

β2-integrins form leukocyte adhesion molecules on the surface of lymphocytes, and could be linked to promoting inflammation in a transplant graft *via* their previously described roles in lymphocyte trafficking and disease activity in IBD. Antibodies against cell adhesion molecules, such as the α4β7 integrin blocker vedolizumab are already used in IBD. Trentadue et al. (38) describe a case of acute rejection after bowel transplant being successfully treated with vedolizumab. Fitzpatrick et al. (17) used single cell RNA sequencing to demonstrate transcriptionally distinct tissue resident T cell subtypes from the donor after bowel transplant. One of these sub-populations highly expressed β2-integrin, the study suggests this may be used to identify tissue resident CD8 cells. Anti-adhesion molecules may be useful in bowel transplant if future studies confirm a role for β2-integrins in the development of graft rejection.

### Proteosome Inhibitors and Purine Analogs

Proteosome inhibition has been shown to reduce incidence of antibody mediated rejection (AMR) (39). AMR is a process of graft rejection driven by plasma cells (derived from B cells) which generate and release immunoglobulin antibodies. The proteosome complex exists in cells to degrade damaged proteins, proteosome inhibitor bortezomib inhibits chymotrypsin activity within the proteosome complex. Inhibition of the proteasome can induce apoptosis of plasma cells, and bortezomib is shown to be effective in clinical studies of AMR treatment post-renal transplant (40). There are case reports describing use bortezomib as a rescue treatment for AMR after bowel transplant (41). Purine analog MMF is already used for long term immunosuppression post-intestinal transplant due to it's ability to inhibit T cell proliferation. Vela et al. (42) described a rat model of intestinal transplant where purine analog fludarabine is used as an induction agent infused into the graft *ex-vivo*. Fudarabine is a purine analog with T cell toxicity currently used in chemotherapy for leukemia. The study concludes this treatment reduced donor cell chimerism, severity of GvHD and improved survival.

## Microbiome Manipulation

The gut microbiome is extensively studied, including profiles in intestinal failure and subsequent gut adaptation alongside parenteral nutrition (43). No therapeutic microbiome strategies have yet been proposed for management of intestinal failure or transplant. Communication between the microbiome and the immune system can modulate immune responses, therefore, microbiome manipulation may have a therapeutic role in intestinal transplant management.

Hartman et al. (44) described that the microbiome in stoma effluent obtained post-small bowel transplant was similar to their control cohort (patients who had ileostomy for other reasons), with a predominance of lactobacilli and enterobacteria. This then reverted to “normal” (as compared to published normative data) after reconnection. Oh et al. (45) subsequently described the microbiota composition of ileal effluent associated with rejection episodes post-small bowel transplant. They demonstrated a different microbial community to Hartman, dominated by *Streptococcus, Escherichia* and *Klebsiella* (as well as Lactobacillus and Enterococcus described by Hartman), and that this significantly changed in episodes of acute rejection with expansion of the *E.coli* and *Klebsiella* species.

Weber et al. (46) undertook a prospective study to measure L-tryptophan metabolites of gut microbiota in urine as a predictor of intestinal GvHD in patients after stem cell transplant. They found that low levels of the metabolite 3-indoxyl sulfate was associated with poor outcomes, hypothesizing that the microbial signature associated with high urinary levels of 3-indoxyl sulfate is suggestive of a more diverse microbiome, and had a bacteriostatic effect on enterococci. The study also showed that higher, possibly protective, levels of metabolite were seen in patients treated with rifaximin for gut decontamination vs. ciprofloxin/metronidazole. Together, these studies suggest microbiome profiling may be developed for use as a marker of allograft rejection in the future.

The success of manipulation of the microbiome to prevent or treat rejection episodes is difficult to assess. Clinical studies have difficulty in demonstrating whether the dysbiosis associated with rejection is cause or consequence. Krams et al. (47) have studied a mouse model of small bowel transplant to demonstrate that TLR4 (Toll-like receptor 4, a cellular receptor known to interact with bacterial lipopolysaccharide to induce inflammatory responses) knock out mice have longer graft survival than their wild type controls. A rat model of intestinal transplant demonstrated a similar rejection-associated dysbiosis to the human studies, and showed that dietary fish oil can normalize this dysbiosis and improve tight junction integrity; potentially resulting in reduced inflammatory cell infiltrate into the graft (48).

There are case reports of the safety of fecal microbiota transplant (FMT) to treat refractory Clostridium difficile colitis in immunosuppressed renal and lung transplant patients (49). A recent multicenter retrospective review including a small number of bowel transplant patients receiving FMT for management of *C. difficile* colitis also demonstrated safety and efficacy but with a risk of CMV reactivation (50).

Whilst studies suggest that profiling the microbiome may be a useful non-invasive marker for rejection, manipulating it to prevent or treat rejection episodes is more difficult to assess, but offers potential for future therapies.

## Tissue Engineering

The holy grail of transplant management is graft tolerance without the need for immunosuppression. Patient-derived grafts would be a way to achieve this. In the meantime, autologous immune cells have the potential to allow reduced immunosuppression by inducing tolerance.

### CAR-T Cell Therapy

Chimeric Antigen Receptor (CAR) T cell therapy is a personalized T cell therapy that has been approved for use in the UK to treat leukemia. T cells are engineered for each patient individually to target cancer cells. Initial studies have been undertaken with autologous regulatory T cell therapy (using similar methodology to CAR T cell expansion) as a possible mechanism to induce tolerance post-liver transplant (51). The studies have described a safe protocol for ongoing research to explore personalized cell based immunomodulation in solid organ transplant.

### Intestinal Organoids

Tissue engineering at an organ level is fast advancing. Intestinal organoids are derived from isolation of stem cells from intestinal crypts using *ex-vivo* intestinal tissue. The stem cells are then cultured *in-vitro* within a matrix and a mixture of growth factors to develop into 3D organoids representative of intestinal epithelium with villi, crypts, functional enterocytes, as well as goblet, Paneth and enteroendocrine cells (52).

Grant et al. have developed tissue engineered small intestine (TESI) derived from human and mouse organoids. These were then implanted into a mouse model where they demonstrated good cell differentiation and polarization within a crypt structure analogous to that of native tissue (53). They were also able to demonstrate expression of secretory and absorptive cell transporters, and *ex-vivo* functional brush border enzymes, suggesting the possibility to develop absorptive capacity. Workman et al. (54) have engineered human stem cell derived intestinal tissue with a functional enteric nervous system *in vitro*, using co-culture of epithelial organoids with neurospheres; potentially a future treatment for long-segment Hirschprung's disease.

Engineered intestinal grafts have been transplanted into rats and demonstrated functionality *in vivo*. Griksheit et al. undertook massive small bowel resection in their rat model and then transplanted tissue engineered small intestine. The transplanted rats showed more rapid post-operative weight gain and serum B12 levels compared to the control group (55). Meran et al. have described the use of patient intestinal mucosa biopsies to develop an organoid, which demonstrated subsequent cell proliferation with maintenance of an intestinal signature and seeding on to a human scaffold. Pig and mouse models were used to demonstrate functional capacity and *in-vivo* survival (56).

## Summary

**Figure 1:**
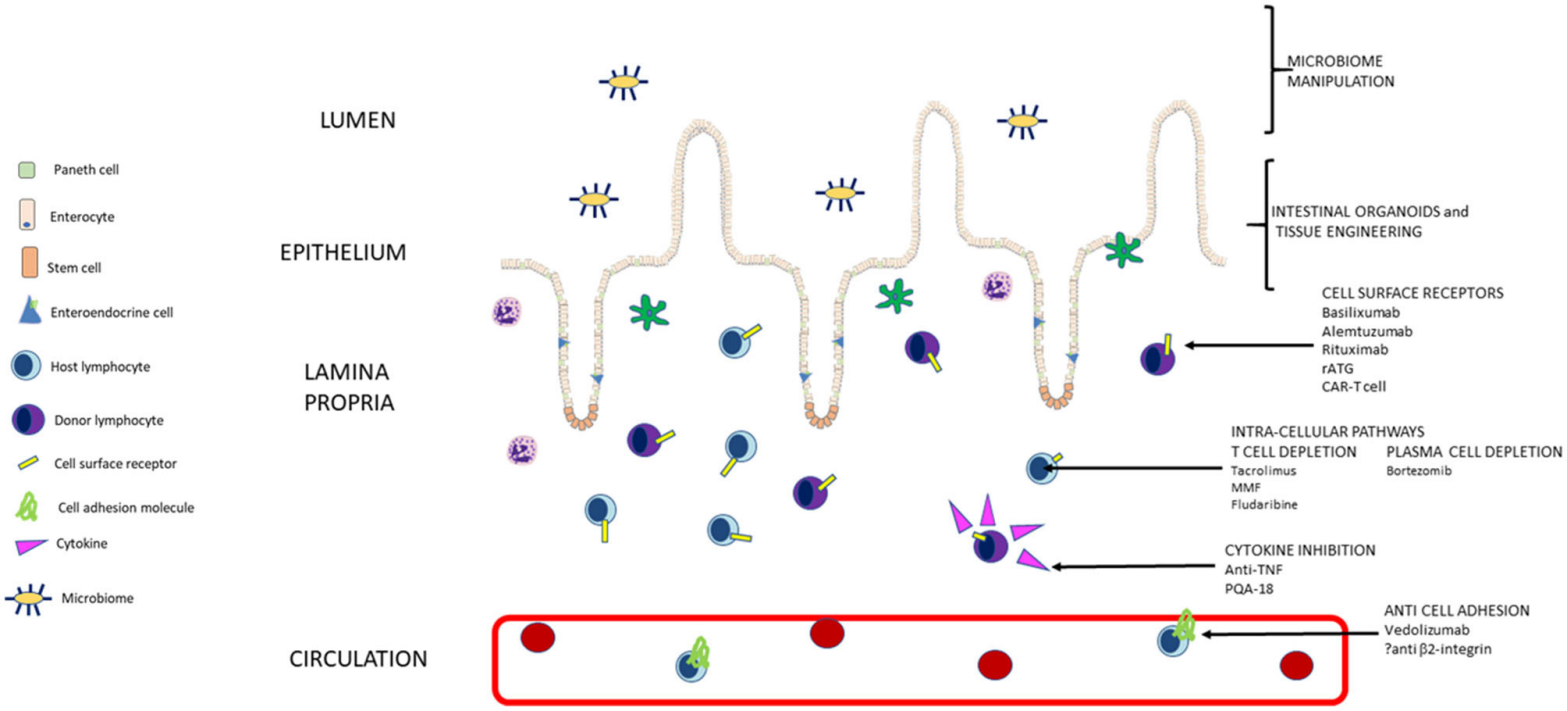
Summary of pathways targeted by current and new approaches to immunosuppression in intestinal transplant (not to scale).

Progress in management of intestinal transplant with the novel therapies discussed offers the exciting prospect of improved graft tolerance and survival with less immunosuppressive drug side effects. With appropriate patient selection, we hope this allows intestinal transplant to be offered pre-emptively, with curative intent for irreversible intestinal failure, rather than after complications of long-term parenteral nutrition have already set in (Figure 1).

## Author Contributions

All authors listed have made a substantial, direct, and intellectual contribution to the work and approved it for publication.

## Conflict of Interest

The authors declare that the research was conducted in the absence of any commercial or financial relationships that could be construed as a potential conflict of interest.

## Publisher's Note

All claims expressed in this article are solely those of the authors and do not necessarily represent those of their affiliated organizations, or those of the publisher, the editors and the reviewers. Any product that may be evaluated in this article, or claim that may be made by its manufacturer, is not guaranteed or endorsed by the publisher.
